# OncoBinder facilitates interpretation of proteomic interaction data by capturing coactivation pairs in cancer

**DOI:** 10.18632/oncotarget.7305

**Published:** 2016-02-10

**Authors:** Samya Van Coillie, Lunxi Liang, Yao Zhang, Huanbin Wang, Jing-Yuan Fang, Jie Xu

**Affiliations:** ^1^ State Key Laboratory for Oncogenes and Related Genes, Key Laboratory of Gastroenterology & Hepatology, Ministry of Health, Division of Gastroenterology and Hepatology, Renji Hospital, School of Medicine, Shanghai Jiao Tong University, Shanghai Institute of Digestive Disease, Shanghai 200001, China; ^2^ Faculty of Medicine, Catholic University Leuven, Leuven, B-3000, Belgium

**Keywords:** Chromosome Section, protein-protein interaction, cancer genome, copy number alteration, gene mutation

## Abstract

High-throughput methods such as co-immunoprecipitationmass spectrometry (coIP-MS) and yeast 2 hybridization (Y2H) have suggested a broad range of unannotated protein-protein interactions (PPIs), and interpretation of these PPIs remains a challenging task. The advancements in cancer genomic researches allow for the inference of “coactivation pairs” in cancer, which may facilitate the identification of PPIs involved in cancer. Here we present OncoBinder as a tool for the assessment of proteomic interaction data based on the functional synergy of oncoproteins in cancer. This decision tree-based method combines gene mutation, copy number and mRNA expression information to infer the functional status of protein-coding genes. We applied OncoBinder to evaluate the potential binders of EGFR and ERK2 proteins based on the gastric cancer dataset of The Cancer Genome Atlas (TCGA). As a result, OncoBinder identified high confidence interactions (annotated by Kyoto Encyclopedia of Genes and Genomes (KEGG) or validated by low-throughput assays) more efficiently than co-expression based method. Taken together, our results suggest that evaluation of gene functional synergy in cancer may facilitate the interpretation of proteomic interaction data. The OncoBinder toolbox for Matlab is freely accessible online.

## INTRODUCTION

Proteins are the main actors in a cell, carrying out an enormous amount of diverse functions, but they rarely act alone. Typically, a protein interacts with different binding partners, often other proteins, to form a molecular complex which allows for various molecular processes to be activated. Because of the significance such protein-protein interactions (PPIs) bring along in the survival and functioning of any living cell, aberrant PPIs are at the source of multiple diseases, including cancer [1, 2]. Therefore it is of great interest to obtain a profound insight in different PPIs and their corresponding function.

Co-immunoprecipitation mass spectrometry (co-IP MS) and yeast two hybrid (Y2H) are the two most widely used techniques in PPI proteomics. While the two-hybrid system mainly identifies direct binary interactions, mass spectrometry can identify the components of a complex, therefore they are considered complementary. Thus, combination of data coming from both approaches allows for a more complete and reliable map of interactions. Although they have definitely proven their worth, they also share similar limitations [3, 4]. Because every aspect of their procedures (reagents used, cell type, experimental conditions, etc.) has a big influence on the proteins detected, the outcomes of different studies are often very heterogeneous and false positives as well as false negatives are a common issues [4, 5]. To date, only a limited fraction of high-throughput PPI data has been functionally annotated in pathway databases such as the Kyoto Encyclopedia of Genes and Genomes (KEGG), or confirmed by low-throughput studies [6]. The high number of false positives makes the interpretation of proteomic interaction data highly challenging [3, 5, 7].

To evaluate the confidence and functional relevance of proteomic interaction data, it is rational to consult other information such as structure-based prediction or co-expression modules [5]. Currently, structure-based prediction (protein-protein docking) is relatively time-consuming and limited by the availability of experimentally determined protein structures [8–14]. Co-expression modules (or gene sets, signatures) inferred from microarray or RNA-sequencing data [15, 16] are often used to infer functionally related genes [17–20]. However, it has been pointed out that co-expression modules don't seem to be reproducible across experiments or capture the functional status in the corresponding studies [21]. Recent cancer genomic studies such as The Cancer Genome Atlas (TCGA) have incorporated other data types such as gene mutations and copy number alterations (CNAs), allowing for a more accurate estimation of gene function and pathway status. Therefore, we proposed that “coactivation pairs” may be inferred from these data and facilitate the functional interpretation of proteomic interactions that have been poorly annotated.

Taking into account the notions above, the OncoBinder tool was developed based on a ranked “coactivation” metric to identify cancer-related PPIs. The functional status of protein-coding genes was estimated by a decision tree model containing three nodes (gene mutation, CNA and expression), and their correlation was used as a measure for identifying coactivation pairs in cancer. Since the EGFR and ERK2 proteins are potential therapeutic targets in gastric cancer, understanding their binding partners represents a highly interesting question. Both proteins have been suggested to associate with a large number of unannotated binders by high-throughput methods, as recorded by the BioGRID database. We applied OncoBinder to identify their “coactivated” binders in gastric cancer, based on the TCGA dataset. The co-expression method was also used, to compare both the techniques accuracy. By these approaches, we aim to test if a “coactivation” metric based on cancer genomic data may be helpful for interpreting high-throughput PPI data.

## RESULTS

### Modeling coactivation of protein partners

**Figure 1:**
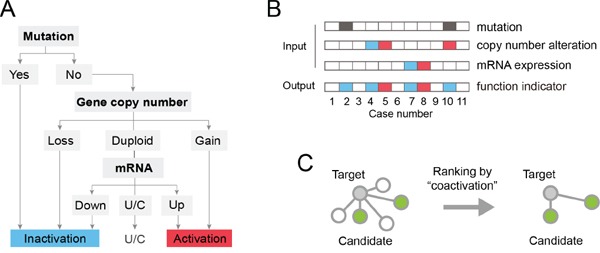
Schematic representation of the OncoBinder model **A.** The decision tree model for defining the functional status of protein-coding genes. The model includes 3 nodes (mutation, gene copy number, mRNA), 6 leaves (end points) and 3 labels (inactivation, activation and unchanged). “U/C” indicates unchanged. **B.** Representative conditions showing the principles of OncoBinder. The dark grey boxes indicates mutation of the gene. The red color represents increased gene copy number or mRNA expression level, or activation in its function. The blue color indicates the opposite conditions (decreased CNA or mRNA expression, or inactivation in function). The gene was labeled as “inactivation” in cases #2 (because of mutation), #4 (decreased CNA), #7 (mRNA donwregulation) and #10 (combination of mutation and increased CNA). The “activation” label was assigned to cases #5 and #8 for increased CNA and mRNA expression, respectively. **C.** A subset of interactions (green nodes) are selected by OncoBinder according to their coactivation patterns in cancer.

We developed a decision tree model to assess the functional status of protein-coding genes, based on cancer genomic data including gene mutation, copy number alteration and mRNA expression (schematic representation in Figure 1A). Three classes of functional status (labels) were defined: activation, inactivation, and unchanged. The decision tree contains three nodes and 6 leaves, and its first node is the genetic status of the gene that encodes the corresponding protein. If the gene is mutated, an inactivation label is assigned. Otherwise, the gene copy number (second node) is evaluated to assign inactivation (gene copy number loss or deletion), activation (copy number gain or amplification), or proceed to the third node(mRNA expression). When the mRNA expression level is among the top 20% of all samples, it is considered activated. Likewise, mRNA expression in the bottom 20% is assigned as inactivation and other conditions are recognized as no change in function. By this decision tree model, the functional status of a protein-coding gene is labeled in each tumor sample as activation (value +1), inactivation (−1) or unchanged (0) (Figure 1B). Then the correlation between each pair of protein-coding genes is calculated and used as a measure of “coactivation”. This evaluation method, named OncoBinder, is applied to analyze the proteomic interaction data generated by MS and Y2H obtained via BioGRID, in order to estimate the likelihood of these interactions in the specified cancer (Figure 1C).

### Assessment of EGFR interactions in gastric cancer

The epithelial growth factor receptor (EGFR) is considered as a therapeutic target for many cancers, including gastric cancer [22–25]. Therefore, it is of importance to comprehensively understand the EGFR-associated signaling pathways and identify EGFR-interacting proteins in cancers. Multiple studies based on high-throughput methods have identified 573 potential binders of EGFR, as summarized in the BioGRID database (listed in Supplementary Table 1). Of note, most of these interactions have not been functionally validated or annotated by pathway databases [6]. Based on the notion that functional interactors may be co-activated in cancer, we evaluated these potential EGFR interactors by OncoBinder and analyzed whether the top hits were more likely to be functionally validated/annotated binders. A set of high confidence EGFR binders, either annotated by KEGG database or confirmed by low-throughput co-IP Western Blot or biological activity experiments, were used to validate the results of OncoBinder (Supplementary Table 1).

**Figure 2:**
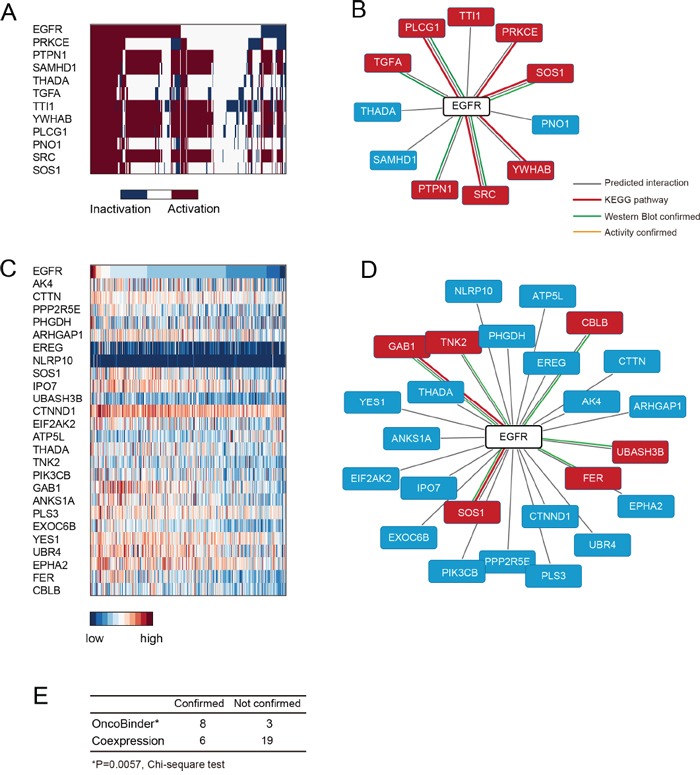
Assessment of EGFR interactions by OncoBinder **A.** Heat map showing the functional status of EGFR binders ranked by their coactivation patterns with EGFR in gastric cancer. Each column represents a cancer case, with colors in dark blue (inactivation) or red (activation) indicating its functional status. **B.** The interactions predicted by OncoBinder, with high-confident interactions highlighted in red (types of evidence in links with different colors). **C.** The genes that are significantly co-expressed with EGFR are ranked according to the significance of correlation. **D.** Binders of EGFR as predicted by co-expression method, with high-confidence interactions shown in red boxes. The colors of linkers indicate the types of evidence supporting the interaction. **E.** Statistical analysis (Chi-square) showing higher accuracy of OncoBinder than co-expression based model.

The OncoBinder algorithm identified 11 binding partners of EGFR that co-activated in gastric cancer (Figure 2A), of which 8were present in the high confidence set (Figure 2B, Supplementary Table 1). This rate of high confidence binders in the OncoBinder-selected set was significantly higher than expected by random selection (P=0.0002, two-sided Chi-square test). Meanwhile, the co-expression method based on mRNA expression suggested 25 binders that significantly associated with EGFR (Figure 2C). However, only 6 of these interactors were supported by KEGG annotation or low-throughput experiments (Figure 2D). Of note, the genes that ranked on top (AK4, CTTN, PPP2R5E, PHGDH, ARHGAP1, EREG, and NLRP10) were not included in the high confidence binder set (Figure 2D), and the accuracy of co-expression displayed no significant difference with random selection (P=0.4806, two-sided Chi-square test). Thus, OncoBinder-based assessment of high-throughput protein interactions seemed to identify high confidence interactions of EGFR more efficiently than co-expression based method (P=0.0057, Figure 2E).

### Evaluation of ERK2 interactions in gastric cancer

**Figure 3:**
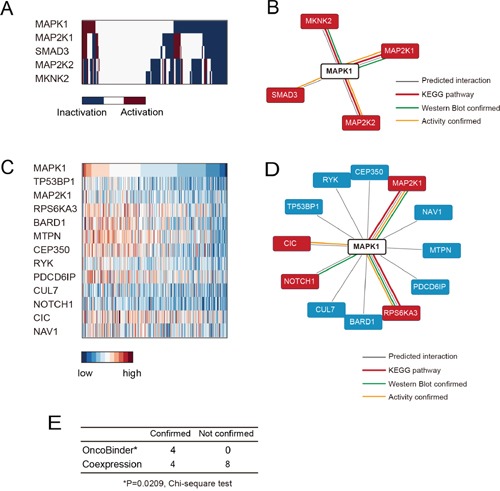
Evaluation of ERK2 interactions by OncoBinder **A.** Heat map showing the functional status of ERK2 interactors in different gastric cancer cases. **B.** The interactions predicted by OncoBinder, with high-confident interactions highlighted in red. The colors of linkers indicate the types of evidence supporting the interaction. **C.** The genes that are significantly co-expressed with ERK2 are ranked according to the significance of correlation. **D.** The binders of ERK2 as predicted by co-expression method, with high-confident binders marked in red. The colors of linkers indicate the types of evidence supporting the interaction. **E.** Statistical analysis (Chi-square) test showing higher accuracy of OncoBinder algorithm than co-expression based model.

The Ras/Raf/Mek/ERK signaling is a significant driver factor in gastric cancer, with a high rate of ERK2 activation/phosphorylation in cancer tissues and cell strains. Therefore, it has been proposed that ERK signaling would be a promising therapeutic target against gastric cancer. Previous studies have suggested that ERK2 phosphorylation is under sophisticated regulation in different cellular compartments, and thus understanding the spatial regulation of ERK2 by different protein interactors would be of interest to the field. We focused on the 52 potential binders of ERK2 suggested by BioGRID database, and applied OncoBinder to evaluate the functional interactions in gastric cancer. Again, the co-expression method was also used as a control (results listed in Supplementary Table 2). Interestingly, all the 4 interactors identified by OncoBinder (MAP2K1, SMAD3, MAP2K2 and MKNK2) were supported by KEGG or low-throughput experiments (Figure 3A&3B), and the rate of high confidence binders was significantly higher than by random selection (P=0.0452, two-sided Chi-square test). Moreover, the co-expression based method suggested 12 binders (Figure 3C), with only 4 in the high confidence binder set (Figure 3D). In fact, the accuracy of co-expression based prediction showed no significant difference than random selection (P=0,3551, two-sided Chi-square test). The OncoBinder algorithm displayed significantly higher accuracy than co-expression based prediction (P=0.0209, Figure 3E).

## DISCUSSIONS

The interpretation of proteomic interaction data is crucial for identifying functional interplay between proteins and signaling pathways. Our work suggests that a coactivation metric derived from cancer omics data may facilitate the identification of functional interactions in cancer, and it was demonstrated that this OncoBinder algorithm displays higher accuracy than co-expression based inference of gene sets or modules.

The improved accuracy may be explained by three features of the OncoBinder method: 1) It recognizes mutations that cause inactivation of the encoded protein, although its expression level may be unaltered; 2) The coactivation metric combines gene CNA information, based on the fact that DNA copy number is a strong influential factor of gene expression and function [26]; 3) A more stringent criterium for defining mRNA upregulation/downregulation may improve the specificity of recognized gene co-expression. These factors, encompassing both the integrality of information and the specificity of analysis, may collectively affect the accuracy of identifying protein interactions in cancer.

To our attention, the top hits provided by OncoBinder were crucial regulators of the EGFR and ERK signaling pathways. The PRKCE (Protein Kinase C, Epsilon) is a key element in EGFR signaling pathway (annotation by KEGG), which promotes the proliferation of gastric cancer cells in response to hypoxia [27]. Its “coactivation” relationship with EGFR as revealed by OncoBinder, is consistent with its crucial roles in EGFR signaling. Moreover, OncoBinder identified MAP2K1 (MEK1) as the top binder of ERK2, and this result fully reflects the central role of MEK1 in phosphorylating and activating ERK2. In contrast, the top hits by co-expression analysis (AK4 for EGFR, and TP53BP1 for ERK2) were not supported by KEGG annotation or low-throughput experiments. These findings, together with the higher rate of high confidence binders in OncoBinder-based analysis, suggest that a coactivation metric is more accurate than co-expression while inferring protein interactions in cancer.

Although the present work demonstrated the assessment of EGFR and ERK2 interactions, the OncoBinder method may be applied to other proteins and their potential binders, found by high-throughput experiments. Although our work may be useful for the interpretation of proteomic interaction data, it may be further improved in the following aspects: Firstly, the estimation of functional status of genes can be combined with other omics information such as DNA methylome (data available in some cancer types), protein expression and phosphorylation. Although currently the protein-level data are still limited to <200 well-studied proteins, future advances in cancer proteomic research may facilitate the simultaneous detection of all proteins in cancer tissues. Such information would be an ideal replacement of mRNA expression data, due to the considerable inconsistence between mRNA and protein expression levels. Secondly, it may be useful to combine Gene Ontology (GO) terms, such as the cellular compartment and recently introduced “biological phase” (denoting a period or stage in cell cycle), since protein interaction is more likely to occur when the partner proteins are localized in the same cellular compartment, or are activated in the same biological phase. While the above-mentioned factors should in theory improve the interpretation of proteomic interaction data, the detailed procedures for their integration to the existing coactivation model certainly require substantial investigation.

## MATERIALS AND METHODS

### Scoring metric for coactivation of protein binders

The functional statuses of genes encoding the protein interactors were estimated according to the mutation, copy number alteration (CNA) and mRNA expression from cancer genomic data. The workflow of OncoBinder is shown in Figure 1. A decision tree containing 3 nodes (mutation, CNA, mRNA), 6 leaves and 3 labels (activation, inactivation, or unchanged) was used to determine the functional status of the protein-coding gene. The types of mutations included missense, nonsense, frameshift, split, insertion and deletion that caused alterations in the amino acid sequence of the coded proteins. When a gene is mutated, it is considered inactivated. With CNA of +1 or +2 a gene is considered as activated, while −1 and −2 in CNA were considered inactivated. When no copy number change is found, the status of the gene is stipulated by its mRNA expression as activated (in top 20%), inactivated (bottom 20%) or unchanged (other). The functional statuses of the genes were represented by numerical values as 1 (activation), 0 (unchanged), or −1 (inactivation). For estimating the coactivation of a pair of proteins, the respective coding genes were analysed by Pearson correlation of their functional status in all cancer samples. The criteria for considering significant correlation was set as P<0.001, and can be adjusted according to the degree of stringency. Only protein-coding genes on different chromosomes were subjected for correlation analysis, because genes located on the same chromosome are prone to be amplified or deleted together without biological significance.

### Pre-processing the input data

The different types of cancer omics data, encompassing mutations, CNAs and mRNA expression, were processed to have the same numbers of genes (rows) and samples (columns). In the present study, the TCGA gastric cancer dataset was downloaded and processed in the following file formats: CNAs (−2, −1 0, +1, or +2) saved as “cnv.xlsx”; mutations (1 for mutation and 0 for wild type) in “mutation.xlsx”; and mRNA expression levels (normalized expression values) in “expression.xlsx”. Note that these different files were also processed to have the same order of genes and samples. The high-throughput protein interaction information was saved in “PPI_high_throughput.xlsx”, with each row denoting one pair of binding partners (official gene symbols). In this study, the protein interaction data were obtained through the BioGRID database, with a filter for selecting only co-IP MS or Y2H results. The data file describing the genomic locations of all investigated genes is saved as “gene_location.xlsx”.

### Setup and execution of the OncoBinder algorithm

The OncoBinder program can be executed in the Matlab computing environment (versions above 2012, The Mathworks, Inc), and different parameters can be adjusted for customizing the study. The target oncoprotein can be edited in the variable input area of the “OncoBinder.m” file, and the default gene name is MAPK1. Note that only official gene symbols can be recognized by the program. Furthermore, the algorithm allows defining of the criteria to outline upregulation (top 20% in default) or downregulation (bottom 20% in default) of mRNA expression. Also, the threshold for statistical significance can be edited if different stringency is applied (default as P<0.001). After the input parameters are defined, the program file should be saved in the working directory of Matlab, together with the input data files: “cnv.xlsx”, “mutation.xlsx”, “expression.xlsx”, and “PPI_high_throughput.xlsx”. The analysis can be started by typing “oncobinder” in the command line of Matlab.

### Acquisition and interpretation of output data

An overview graph is generated to show the correlation between the functional status of the target protein (first row) and its putative binders (ranked by significance of association). The output of EGFR analysis is presented in Figure 2A, with activation marked in dark red and inactivation in blue. The relationship between different protein-coding genes can be directly observed in this plot. Moreover, the names of ranked binders can be found in the “order_names” variable in the Matlab workspace and exported to EXCEL program. Likewise, the functional status and the significance (P-values of Pearson correlation) of the ranked binders are stored in the “order_status” and “order_corr” variables in the Matlab workspace.

## SUPPLEMENTARY TABLES
















