# Supplementary material for: OncoBinder facilitates interpretation of proteomic interaction data by capturing coactivation pairs in cancer
Source: Oncotarget. 2016 Feb 10;7(14):17608–15. doi: 10.18632/oncotarget.7305 (PMC4951236; doi:10.18632/oncotarget.7305)
Supplement: Supplementary file 1 [file oncotarget-07-17608-s001.pdf]

SUPPLEMENTARY TABLES

Supplementary Table 1: Binders of EGFR predicted by OncoBinder or Coexpression-based algorithms

See Supplementary File 1

Supplementary Table 2: Binders of MAPK1 predicted by OncoBinder or Coexpression-based algorithms

See Supplementary File 2
